# Heat‐shock transcription factor 1 is critically involved in the ischaemia‐induced cardiac hypertrophy via JAK2/STAT3 pathway

**DOI:** 10.1111/jcmm.13713

**Published:** 2018-07-11

**Authors:** Lingyan Yuan, Lin Qiu, Yong Ye, Jian Wu, Shuchun Wang, Xingxu Wang, Ning Zhou, Yunzeng Zou

**Affiliations:** ^1^ Department of kinesiology Institute of physical education Shanghai Normal University Shanghai China; ^2^ Department of Pharmacy Tongji Hospital Tongji Medical College Huazhong University of Science and Technology Wuhan China; ^3^ Shanghai Institute of Cardiovascular Diseases Zhongshan Hospital and Institutes of Biological Science Fudan University Shanghai China; ^4^ Department of Computer Tomography and Magnetic Imaging Yidu Central Hospital Weifang Medical College Weifang China; ^5^ Division of Cardiology Department of Internal Medicine Tongji Hospital Tongji Medical College Huazhong University of Science and Technology Wuhan China

**Keywords:** cardiac hypertrophy, heat‐shock transcription factor 1, Janus kinase 2, myocardial infarction, signal transducer and activator of transcription 3

## Abstract

Cardiac hypertrophy after myocardial infarction (MI) is an independent risk factor for heart failure. Regression of cardiac hypertrophy has emerged as a promising strategy in the treatment of MI patients. Here, we have been suggested that heat‐shock transcription factor 1 (HSF1) is a novel repressor of ischaemia‐induced cardiac hypertrophy. Ligation of left anterior descending coronary was used to produce MI in HSF1‐deficient heterozygote (KO), HSF1 transgenic (TG) mice and their wild‐type (WT) littermates, respectively. Neonatal rat cardiomyocytes (NRCMs) were treated by hypoxia to mimic MI in vitro. The HSF1 phosphorylation was significantly reduced in the infarct border zone of mouse left ventricles (LVs) 1 week after MI and in the hypoxia‐treated NRCMs. HSF1 KO mice showed more significant maladaptive cardiac hypertrophy and deteriorated cardiac dysfunction 1 week after MI compared to WT MI mice. Deficiency of HSF1 by siRNA transfection notably increased the hypoxia‐induced myocardial hypertrophy in NRCMs. Mechanistically, Janus kinase 2 (JAK2) and its effector, signal transducer and activator of transcription 3 (STAT3) were found to be significantly increased in the LV infarct border zone of WT mice after MI as well as the NRCMs treated by hypoxia. These alterations were more significant in HSF1 KO mice and NRCMs transfected with HSF1 SiRNA. Inversely, HSF1 TG mice showed significantly ameliorated cardiac hypertrophy and heart failure 1 week after LAD ligation compared to their WT littermates. Our data collectively demonstrated that HSF1 is critically involved in the pathological cardiac hypertrophy after MI via modulating JAK2/STAT3 signalling and may constitute a potential therapeutic target for MI patients.

## 1 INTRODUCTION

As a major cause of cardiovascular morbidity and mortality, myocardial infarction (MI) has drawn extensive attention for decades.^1^ Post‐MI cardiac hypertrophy is an independent risk factor of sudden cardiac death and heart failure, which has emerged as a promising target in the treatment of patients with MI.^2^ The underlying molecular mechanisms of cardiac hypertrophy have not fully elucidated yet; thus, effective strategies mitigating the cardiac hypertrophy are still far from the attainment of clinical cure throughout the last decades. Therefore, a better understanding of the molecular mechanisms regulating the hypertrophy‐related signalling pathways is important for the development of new therapies to treat pathological cardiac hypertrophy and heart failure after MI.

Heat‐shock transcription factor 1 (HSF1) is a transcription factor of the heat‐shock genes activated after temperature stress.^3^ HSF1 exists in cytoplasm as an inactive monomer in a complex with HSP40/HSP70 and HSP90.^4^ Upon stress, phosphorylation of HSF1 on Ser230 positively contributes to the transcriptional activity of HSF1, which was trimerized after dissociating from the chaperone complex.^5^ It was demonstrated as a key factor involved in the adaptive mechanism of transition from adaptive cardiac hypertrophy to maladaptive heart failure.^6^ Our previous studies indicated that HSF1 functions as an essential intrinsic cardioprotective factor attributing to decreases of pathological cardiac hypertrophy and myocardial fibrosis against ischaemia injury and pressure overload.^7^, ^8^ HSF1 inhibits the pressure overload‐induced cardiac hypertrophy and thus maintains the cardiac function through the interaction with signal transducer and activator of transcription 3 (STAT3).^7^ STAT3, together with its upstream regulator, Janus kinase 2 (JAK2), has been proved to play detrimental roles in the cardiac hypertrophy and the transition to heart failure.^9^, ^10^ As increasing evidence illustrated that HSF1 is a potential therapeutic target of cardiac hypertrophy, we here have been suggested that by targeting JAK2/STAT3 signalling pathway, HSF1 may open new pharmacological venues for treating cardiac hypertrophy and heart failure after MI to supplement current drugs that target the sympathetic or renin‐angiotensin systems.

In this study, using LAD ligation in HSF1 KO and TG mice, together with hypoxic treatment in NRCMs infected with adenovirus of HSF1 SiRNA, we uncovered that deficiency of HSF1 deteriorated cardiac hypertrophy and cardiac dysfunction after MI through enhancing the activity of JAK2/STAT3 signalling. Whereas enhanced HSF1 activation inhibited the cardiac hypertrophy after MI in HSF1 TG mice. Our findings may provide an optional therapeutic strategy to inhibit pathological cardiac remodelling and improve the cardiac function after MI.

## 2 MATERIALS AND METHODS

### 2.1 Isolation of neonatal rat cardiomyocytes

Primary cultures of neonatal rat cardiac myocytes were prepared as described previously.^8^, ^11^ Briefly, 1‐ to 2‐day‐old Sprague Dawley rats were killed by swift decapitation and their hearts were immediately removed under aseptic conditions and placed in ice‐cold sterile PBS (calcium‐ and magnesium‐free). Enzymatic and mechanical dissociation of cardiomyocytes was then performed using the Neonatal Cardiomyocyte Isolation System supplied by Worthington Biochemicals (USA). The hearts were minced on ice and digested 15 minutes for 7‐8 times with purified trypsin (10 μg/mL) in PBS at 37°C with intermittent gentle swirling. Mild trituration was used to dissociate the digested tissue mechanically, and single‐cell suspensions were obtained by filtering this digested material through 70 μm sterile mesh filters. The cells were collected by low‐speed centrifugation (1500 rpm for 10 minutes at room temperature). The supernatant was discarded and the cell pellet resuspended in DMEM (high glucose) culture medium containing 10% FCS (Hyclone Laboratories, USA). Dispersed cells were pre‐plated for 90 minutes to remove fibroblasts and other proliferation cells, and unattached cells counted and seeded onto 6‐well culture plates at a density of 1 × 10^6^/well. The media was changed every 48 hours beginning the day after seeding. Bromodeoxyuridine (0.1 mmol/L) was added to the culture media for the first 72 hours to minimize contamination from fibroblasts. Using this method, we routinely obtained primary cultures with >95% myocytes, as assessed by microscopic observation of spontaneous contraction and by immunocytochemical staining with a monoclonal anticardiac α‐myosin heavy‐chain antibody.

### 2.2 Treatment of neonatal rat cardiomyocytes

We used replication‐defective adenoviral vectors encompassing the entire coding region of the HSF1 gene (shHSF1) under the control of the cytomegalovirus promoter. A similar adenoviral vector encoding the green fluorescent protein (GFP) gene was used as a control. To knock down HSF1 expression, shHSF1 was commercially purchased from Santa Cruz Biotechnology, Inc. Next, we generated 3 shHSF1 adenoviruses (AdshHSF1) and selected the one that produced a significant decrease in HSF1 levels for further experiments. AdshRNA was used as the non‐targeting control. We infected cells with AdshHSF1 and AdshRNA at a MOI of 100, which resulted in transgene expression without toxicity in 95%‐100% of the cells. Seventy‐two hours after siRNA transfection, myocytes were further cultured under either normoxic (37°C, 5% CO_2_) or hypoxic (37°C, 1.5% O_2_) condition for 24 hours.

### 2.3 Immunofluorescent staining of cardiomyocytes

Immunofluorescent staining for NRCMs was performed using anti‐α‐MHC) primary antibody and 4′,6‐Diamidino‐2‐Phenylindole, Dihydrochloride (DAPI) staining for 30 minutes minutes as described previously.^12^ Myocyte surface area from at least 100 cells per group in each experiment were analysed using the ImageJ program.

### 2.4 Animal model

The generations of HSF1‐deficient heterozygote (KO) mice and HSF1 transgenic (TG) mice have been described previously.^8^ HSF1 KO and TG mice and their respective wild‐type littermates (WT) (aged from 8 to 10 weeks) were used in this study. The mice were subjected to the LAD coronary ligation or sham operation as described previously.^7^ Briefly, the mice were sedated with 2% isoflurane inhalation. Then the mouse pre‐cordial chest was incised between the 3^rd^ and 4^th^ rib to expose the heart after intubation and ventilation. 10/0 proline suture was passed under the LAD and ligated doubly. Finally, the chest wall was closed. Sham operation was performed by following the same procedure without LAD ligation. All animal experimental protocols were approved by the Animal Care and Use Committee of Fudan University and in compliance with “Guide for the Care and Use of Laboratory Animals (the Guide, NRC 2011).”

### 2.5 Echocardiographic measurements

Echocardiography and invasive haemodynamic measurement were performed at baseline and 1 week after sham or LAD ligation surgery. Mice were anesthetized by inhalation of 2% isoflurane. Transthoracic echocardiographic analysis was performed using an animal‐specific instrument (VisualSonics Vevo770, VisualSonics Inc., Canada) as previously described.^8^, ^11^ Mice were anesthetized and M‐mode images of the left ventricle (LV) were recorded. Cardiac structure and function parameters included left ventricular internal end‐diastolic dimensions (LVIDd), left ventricular anterior wall end‐diastolic thickness (LVAWd) and left ventricular ejection fraction (LVEF). All measurements were averaged for 5 consecutive cardiac cycles and were carried out by 3 experienced technicians who were unaware of the identities of the respective experimental groups.

### 2.6 Invasive haemodynamic test

Haemodynamic assessment was performed by a 1.4 F pressure catheter (SPR 671, Millar Instruments) inserted into the aorta and LV through the right common carotid artery after mice were anesthetized by inhalation of 2% isoflurane.^8^, ^11^ The transducer was connected to Powerlab system (AD Instruments, Castle Hill, Australia). Cardiac morphological and haemodynamic parameters including LV end‐systolic pressure (LVESP), LV end‐diastolic pressure (LVEDP) and maximal contraction and relaxation velocity (max d*p*/d*t* and min d*p*/d*t*) were record and analysed.

### 2.7 Pathological test

One week after the LAD ligation, mice were killed and hearts were excised. After dissecting LV, part of myocardial samples was snap‐frozen with liquid nitrogen for protein as well as mRNA analysis and the other part was fixed for 24 hours in 4% formalin dissolved in 0.1 mol/L PBS (pH 7.4), subsequently embedded in paraffin and transversely cut into 5 μm sections onto slides for further histological analysis. The myocyte cross‐sectional area (CSA) was measured with a quantitative digital image analysis system (Image‐Pro Plus 6.0) using images that were captured from FITC‐conjugated wheat germ agglutinin (WGA, Invitrogen Corp) stained sections as described previously.^12^, ^13^ More than 100 myocytes in the examined sections were outlined for each group of mice. The myocardial apoptosis was measured by terminal deoxynucleotidyl transferase dUTP nick end labelling (TUNEL) according to the manufacturer's instructions (Roche Applied Science, South San Francisco, California, USA). Five micrographs were randomly selected and the numbers of healthy or apoptotic cardiomyocytes were counted. The extent of cell apoptosis was expressed as the ratio of TUNEL‐positive nuclei over DAPI‐stained nuclei. Myocardial infarction size was determined by morphometric analysis as described previously.^7^

### 2.8 Quantitative real‐time PCR

Total RNA was prepared from left ventricular tissues of mouse hearts or cultured NRCMs using TRIzol reagent (Invitrogen, catalogue 15596‐018, USA). cDNA was synthesized from 1 mg RNA of each sample using the TOYOBO ReverTra Ace‐α‐RT‐PCR kit according to the manufacturer`s instruction. Real‐time PCR was performed on a Bio‐Rad IQ5 multicolour detection system using synthesized cDNA. A comparative CT method was used to determine relative quantification of special RNA levels. All real‐time PCRs were performed at least in triplicates. Atrial natriuretic peptide (*ANP*), B‐type natriuretic peptide (*BNP*) and glyceraldehyde‐3‐phosphate dehydrogenase (*GAPDH*) were amplified using their specific primers (Table S1).

### 2.9 Western blotting

Total ventricular extracts were prepared from the LV myocardium as described previously.^14^ The cytoplasmic and nuclear protein was extracted as previously described.^8^ Protein concentrations were measured by BCA (Sigma‐Aldrich, USA) assay. Equal amount of protein extracts was separated with SDS‐page and transferred to a nitrocellulose membrane (Millipore, USA). GAPDH was used for a loading control of total protein. The blots were analysed and quantified by densitometry using ImageJ program.

### 2.10 Statistical analysis

Data are shown as mean ± SEM. Multiple group comparison was performed by one‐way or two‐way ANOVA followed by LSD procedure for comparison of means. Comparison between 2 groups under identical conditions was performed by the 2‐tailed Student's *t* test. A value of *P* < .05 was considered statistically significant.

## 3 RESULTS

### 3.1 Phosphorylation of HSF1 was repressed by both MI in vivo and hypoxic stimuli in vitro

**Figure 1:**
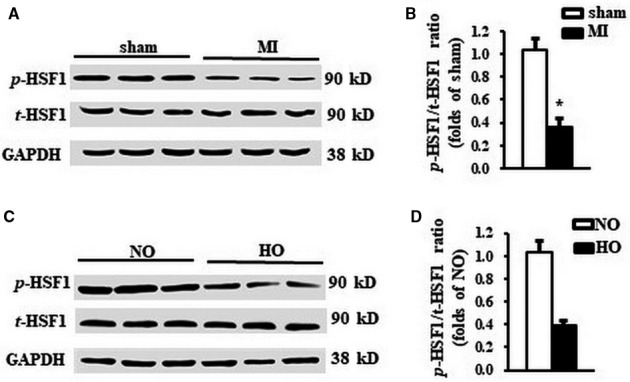
The phosphorylation of HSF1 was reduced by both MI and hypoxic stimuli. A, Representative Western blots and (B) quantitative results of total and phosphorylation level of HSF1 in the LV border zone of MI 1 wk after LAD ligation. N = 5 per experimental group. MI, myocardial infarction. *P < .05 compared with sham. C, Representative Western blots and (D) quantitative results of total and phosphorylation level of HSF1 in NRCMs stimulated by hypoxia. N = 5 independent experiments. GAPDH was served as internal control. NO, normoxia. HO, hypoxia. All data are expressed as mean ± SEM *P < .05 compared with NO

To identify the link between HSF1 and ischaemia‐related cardiac hypertrophy, we measured the expression and phosphorylation of HSF1 in cardiomyocytes in vivo and in vitro. First, we tested the expression and phosphorylation of HSF1 in the infarct border zone of mouse LVs 1 week after MI so as to uncover the changes of HSF1 in the early stage of MI. One week after left anterior descending (LAD) coronary ligation, although the total expression of HSF1 was unchanged, the phosphorylation of HSF1 was found to be significantly decreased by 64% in the infarct border zone area of mouse LV compared to sham mice, respectively (Figure 1A, B). We also measured the expression and phosphorylation of HSF1 in other organs including brain, lung, liver and muscle after MI. However, no significant changes were found before and after MI in the above mouse organs (Figure S1A). Second, to eliminate the possible compensatory or neuroendocrine factors which may influence the expression and phosphorylation of HSF1 in MI‐induced cardiac hypertrophy, NRCMs were isolated and stimulated with hypoxia, a well‐controlled experimental setting to mimic MI in an in vitro study. Similar to the finding from in vivo study, the phosphorylation level of HSF1 was decreased by 72% in the hypoxia‐treated NRCMs compared to the normoxia‐treated NRCMs, respectively (Figure 1 C, D). Taken together, the negative correlation between the HSF1 phosphorylation and hypertrophic response to ischaemia revealed that HSF1 may be critically involved in the pathological process of cardiac remodelling after MI.

### 3.2 Deficiency of HSF1 aggravated hypoxia‐induced myocardial hypertrophy in vitro

**Figure 2:**
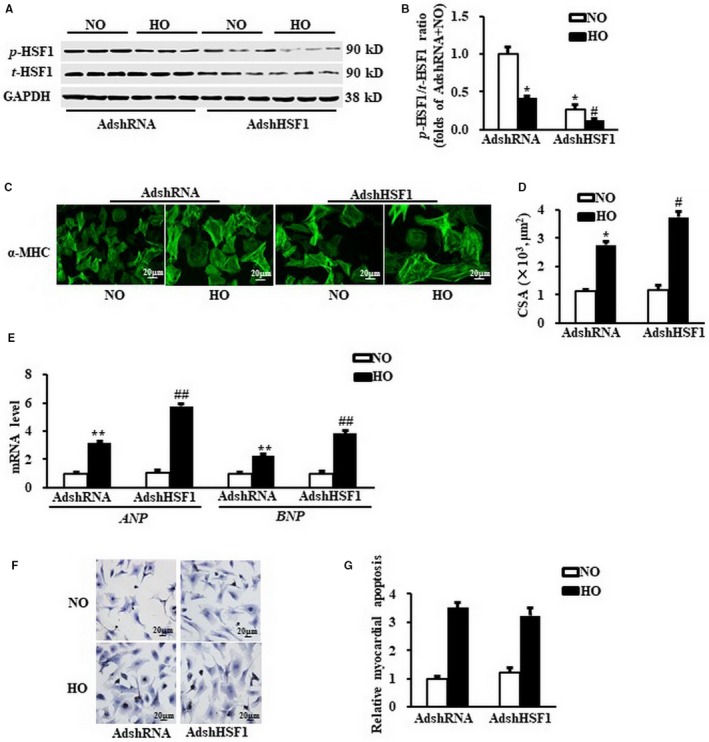
Deficiency of HSF1 deteriorated hypoxia‐induced myocardial hypertrophy in vitro. A,. Representative Western blots of total and phosphorylation level of HSF1 in neonatal cardiomyocytes (NRCMs) after transduced with indicated SiRNA and treated with hypoxia. N = 5 independent experiments. B,. Quantitative results of total and phosphorylation level of HSF1in NRCMs. C and D,. The representative images and quantification data of cardiomyocyte surface area (CSA) of NRCMs stained by α‐myosin heavy chain (α‐MHC). Scale bar: 20 μm. E, mRNA levels of hypertrophic markers, atrial natriuretic peptide (*ANP*), B‐type natriuretic peptide (*BNP*) in NRCMs measured by real‐time PCR. GAPDH was served as internal control. F and G,. The TUNEL staining and quantified data of NRCMs. Scale bar: 20 μm. N = 5 independent experiments. NO, normoxia. HO, hypoxia. All data are expressed as mean ± SEM *P < .05 compared with NO + AdshSiRNA, ^#^ *P* < .05 compared with HO + AdshSiRNA

Given the reduced HSF1 phosphorylation in hypertrophic hearts under myocardial ischaemia, we next determined whether a decrease in HSF1 phosphorylation promotes hypoxia‐induced cardiac hypertrophy. NRCMs were exposed in hypoxic condition for 24 hours to mimic MI in vitro. AdshHSF1 was infected in NRCMs to artificially restrain the expression of HSF1 in the cardiomyocytes (Figure 2A, B). Treatment with hypoxia has no effect on the expression of total HSF1, but significantly inhibited the phosphorylation of HSF1(Figure 2A, B). The NRCMs were then subjected to immunostaining using α‐MHC primary antibody as shown in Figure 2C. Hypoxia increased the cardiomyocyte surface area (CSA) of NRCMs by 2.8‐folds (Figure 2D), while the mRNA level of *ANP* and *BNP,* the cardiac hypertrophic markers, was increased by 3.2‐folds and 2.3‐folds, respectively (Figure 2E). Treatment of AdshHSF1 worsened the enlargement of NRCMs and the up‐regulation of *ANP* and *BNP* (Figure 2D, E). TUNEL assay was performed to detect the myocardial apoptosis. There was an increase in 2.8‐folds in the ratio of cellular apoptosis in hypoxia‐treated NRCMs compared to normoxia‐treated NRCMs. This hypoxia‐induced increase in myocardial apoptosis was similar in the NRCMs treated with AdshHSF1 (Figure 2F). Taken together, deficiency of HSF1 deteriorated myocardial hypertrophy provoked by hypoxia in vitro, indicating a protective role of HSF1 in the hypoxia‐induced myocardial hypertrophy.

### 3.3 Deficiency of HSF1 deteriorated cardiac hypertrophy after MI in vivo

We next explored whether the phosphorylation of HSF1 is necessary for the cardiac hypertrophy induced by MI. First, cardiac morphology and function of the mice were measured by echocardiography 1 week after LAD ligation. The quantitated data showed that there was no significant difference between the HSF1 KO mice and their WT littermates in terms of cardiac morphology and contractile function on baseline level (Table 1), indicating that HSF1 deficiency did not affect cardiac development and growth under physiological condition. One week after MI, WT mice developed a significant left ventricular hypertrophy compared to sham mice, represented by a remarkable increase in LV wall thickness (left ventricular anterior wall end‐diastolic thickness, LVAWd), a notable decrease in left ventricular ejection fraction (EF) as well as a preserved left ventricular internal end‐diastolic dimensions (LVIDd) and heart rate (HR) (Table 1). In addition, MI‐induced haemodynamic alteration was measured by invasive cardiac catheter. Significant decrease in LV end‐systolic pressure (LVESP) and increase in LV end‐diastolic pressure (LVEDP) were observed in MI mice compared to sham mice. Compared to WT MI mice, a significant decrease in the EF and LVESP as well as a notable increase in LVIDd and LVEDP was found in the HSF1 KO mice (Table 1). Furthermore, MI mice also showed a significant decrease in maximal contraction and relaxation velocity (max d*p*/d*t* and min d*p*/d*t*) vs sham. These haemodynamic alterations were more significant in HSF1 KO mice 1 week after LAD ligation (Table 1).

**Table 1:** Echocardiographic and haemodynamic analysis of mice 1 wk after sham or LAD ligation

	Sham		MI	
	WT (n = 8)	HSF1 KO (n = 8)	WT (n = 8)	HSF1 KO (n = 7)
LVAWd (mm)	0.71 ± 0.03	0.73 ± 0.03	1.15 ± 0.06^b^	1.19 ± 0.05
LVIDd (mm)	3.01 ± 0.1	3.10 ± 0.2	3.88 ± 0.3^b^	4.14 ± 0.2^c^
EF (%)	73.8 ± 3.9	74.1 ± 5.7	55.2 ± 2.2^b^	44.5 ± 3.3^c^
HR (bpm)	559.2 ± 49.4	558.5 ± 59.2	574.2 ± 45.2	581.2 ± 46.2
LVESP (mm Hg)	98.6 ± 6.7	97.6 ± 5.8	86.9 ± 3.5^b^	79.6 ± 2.7^c^
LVEDP (mm Hg)	1.5 ± 0.2	1.9 ± 0.2	7.9 ± 0.5^b^	10.8 ± 0.7^c^
Max d*p*/d*t* (mm Hg/S)	10556 ± 579.5	9906 ± 401.4	6908 ± 375.7^a^	5287 ± 445.3^c^
Min d*p*/d*t* (mm Hg/S)	−9026 ± 351.6	−9241 ± 501.6	﹣5511 ± 398.9^a^	−4441 ± 319.8^c^

LVAWd, left ventricular anterior wall end‐diastolic thickness; EF, ejection fraction; LVIDd, left ventricular internal end‐diastolic dimensions; HR, heart rate; LVESP, LV end‐systolic pressure; LVEDP, LV end‐diastolic pressure; max d*p*/d*t*, maximal contraction velocity; min d*p*/d*t*, maximal relaxation velocity.

a *P* < .05.

b *P* < .01 vs. WT sham mice.

c *P* < .05 vs. WT MI mice.

**Figure 3:**
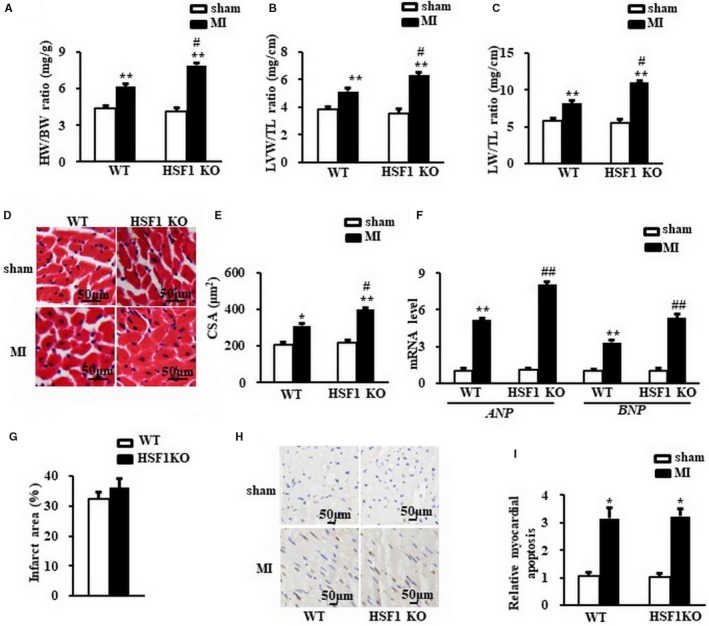
Deficiency of HSF1 aggravated cardiac hypertrophy induced by MI in mice. A, Heart weight/body weight ratio (HW/BW) (B) left ventricular weight/tibia length ratio (LVW/TL) and (C) lung weight/tibia length ratio (LW/TL) of mice. D, Representative images of histological sections of the mouse LVs were stained with haematoxylin‐eosin (H&E) 1 wk after MI or sham surgery. Scale bar: 50 μm. E, Quantitative results of the cross‐sectional area (CSA) of mouse cardiomyocytes quantified using an image analysis system. F,. Real‐time PCR analysis of *ANP* and *BNP* in mouse border zone area of LVs 1 wk after MI. G, the infarction size of the mouse hearts. H and I, The TUNEL staining and quantified data of mouse hearts. Scale bar: 20 μm. GAPDH was served as internal control. N = 7‐8 per experimental group. All data are expressed as mean ± SEM *P < .05, **P < .01 compared with WT sham, ^#^ *P* < .05, ^##^ *P* < .01 compared with WT MI

Cardiac hypertrophy was further determined by the direct ex vivo measurements and histological analysis in the heart tissues 1 week after MI. Compared to sham mice, MI mice exhibited a significant increase in heart weight/body weight ratio (HW/BW) (Figure 3A), LV weight/tibial length ratio (LVW/TL) (Figure 3B), lung weight/tibia length (LW/TL) (Figure 3C) and cross‐sectional area (CSA) of cardiomyocytes (Figure 3D, E). HSF1 KO MI mice showed more significant changes in these direct measurements vs WT MI mice (Figure 3A‐D). Additionally, elevated *ANP* and *BNP* in MI mice vs sham mice were significantly increased in HSF1 KO mice (Figure 3F). To reveal whether the deterioration of cardiac hypertrophy was a resultant change in the myocardial infarction, we measured the infarct area of the mouse hearts. As shown in Figure 3G, HSF1 KO mice showed similar infarct area 1 week after MI compared to WT mice. The deficiency of HSF1 had no significant effect on the myocardial apoptosis in the LV border zone of MI mice (Figure 3H, I). Collectively, these in vivo data, consistent with the observations in vivo, further supported that HSF1 is necessary for the development of cardiac hypertrophy and the transition to heart failure in the early stage of MI.

### 3.4 Deficient HSF1 enhanced the activation of JAK2/STAT3 signalling pathway

**Figure 4:**
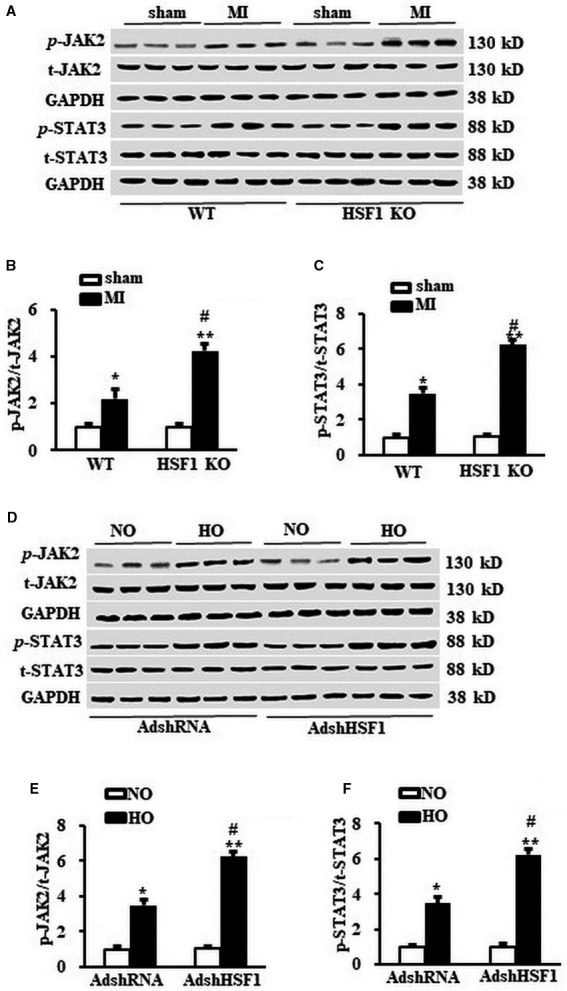
HSF1 regulated the JAK2/STAT3 signalling pathway. A, Representative Western blots and (B) and (C) the quantitative results of total and phosphorylation level of JAK2 and STAT3 in the border zone area of mouse left ventricles (LVs) 1 wk after MI or sham surgery. N = 5 independent experiments in all groups. *P < .05, **P < .01 vs WT sham, ^#^ *P* < .05 vs WT MI. D, Representative Western blots and (E) and (F) the quantitative result of total and phosphorylation level of JAK2 and STAT3 in NRCMs treated by hypoxia and indicated SiRNA. N = 5 per experimental group. All data are expressed as mean ± SEM **P < .01 compared with NO + AdshRNA, ^#^ *P* < .05, ^##^ *P* < .01 compared with HO + AdshRNA

As JAK2 is critically involved in the cardiac hypertrophy, we measured the phosphorylation of JAK2 in both the MI mouse hearts and the hypoxia‐treated NRCMs. We observed that 1 week after MI, the phosphorylation of JAK2 in the infarct border zone of the LV was strikingly increased in the HSF1 KO mice compared with those hearts of the WT mice (Figure 4A, B). Similarly, the phosphorylation of JAK2 in hypoxia‐treated NRCMs was boosted by 2.9‐folds in the NRCMs treated by AdshHSF1 compared with the control NRCMs (Figure 4D,E). STAT3 is a key downstream effector of JAK2. We here tested whether the HSF1 deficiency could increase the activity of STAT3 evoked by ischaemia. As shown in Figure 4A, C, increased phosphorylation of STAT3 was also enhanced in the border zone of the LVs of HSF1 KO mouse in vivo. We also found that the phosphorylated STAT3 was significantly increased in the hypoxia‐treated NRCMs, which were enhanced by the deficiency of HSF1 (Figure 4D, F). These findings collectively demonstrated that ablation of HSF1 increased the activation of JAK2/STAT3 signalling pathway.

### 3.5 Enhanced phosphorylation of HSF1 relieved pathological cardiac hypertrophy induced by LAD ligation in mice

As the deficient HSF1 resulted in a deteriorated cardiac hypertrophy and heart failure, we next explored whether an enhanced phosphorylation of HSF1 abrogates the maladaptive cardiac hypertrophy induced by MI in HSF1 TG mice. The basal cardiac morphology and contractile function were similar between the HSF1 TG mice and their WT littermates through the measurement of echocardiography (Table 2), indicating a normal cardiac development and growth in HSF1 TG mice. The HSF1 TG mice showed significant attenuation of pathological cardiac hypertrophy evidenced by preserved HR, decreased LVAWd, LVIDd and increased EF 1 week after MI compared to WT mice (Table 2). Similarly, haemodynamic abnormalities caused by 1‐week MI in WT mice were notably mitigated in HSF1 TG mice, implying a improvement of contractile function of LV led by HSF1 overexpression (Table 2).

**Table 2:** The echocardiographic and haemodynamic analysis of HSF1 TG mice and their littermates 1 wk after sham or LAD ligation

	Sham		MI	
	WT (n = 6)	HSF1 TG (n = 6)	WT (n = 6)	HSF1 TG (n = 6)
LVAWd (mm)	0.73 ± 0.02	0.72 ± 0.02	1.18 ± 0.05^b^	1.16 ± 0.06
LVIDd (mm)	3.06 ± 0.3	3.03 ± 0.4	3.79 ± 0.5^a^	3.19 ± 0.4^c^
EF (%)	74.3 ± 5.9	75.8 ± 6.1	56.6 ± 4.1^b^	67.9 ± 6.1^c^
HR (bpm)	577.3 ± 47.3	571.0 ± 41.9	585.2 ± 49.5	579.5 ± 49.8
LVESP (mm Hg)	101.5 ± 8.3	103.8 ± 7.5	84.8 ± 6.5^b^	95.6 ± 9.1^c^
LVEDP (mm Hg)	1.4 ± 0.3	1.8 ± 0.4	9.1 ± 0.8^b^	5.1 ± 0.4^c^
Max d*p*/d*t* (mm Hg/S)	9959 ± 661.7	10011 ± 698.8	7002 ± 642.1^a^	8797 ± 660.5^c^
Min d*p*/d*t* (mm Hg/S)	−9261 ± 790.8	−9511 ± 540.1	−5232 ± 577.3^a^	−6719 ± 541.2^c^

LVAWd, left ventricular anterior wall end‐diastolic thickness; EF, ejection fraction; LVIDd, left ventricular internal end‐diastolic dimensions; HR, heart rate; LVESP, LV end‐systolic pressure; LVEDP, LV end‐diastolic pressure; max d*p*/d*t*, maximal contraction velocity; min d*p*/d*t*, maximal relaxation velocity.

a *P* < .05.

b *P* < .01 vs. WT sham mice.

c *P* < .05 vs. WT MI mice.

**Figure 5:**
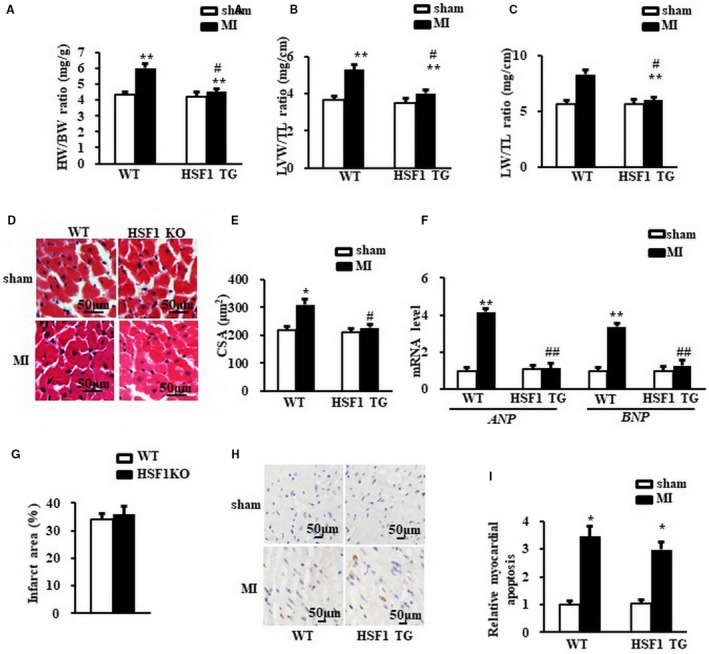
Enhance of HSF1 attenuated cardiac hypertrophy induced by MI in mice. A, Heart weight/body weight ratio (HW/BW), (B) left ventricular weight/tibia length ratio (LVW/TL) and (C) lung weight/tibia length ratio (LW/TL) of mice. D, Representative images of histological sections of the mouse LVs were stained with haematoxylin‐eosin (H&E) 1 wk after MI or sham surgery. Scale bar: 50 μm. E, Quantitative results of the cross‐sectional area (CSA) of mouse cardiomyocytes quantified using an image analysis system. F, Real‐time PCR analysis of *ANP* and *BNP* in mouse border zone area of LVs 1 wk after MI. (G) The infarction size of the mouse hearts. H and I, The TUNEL staining and quantified data of mouse hearts. Scale bar: 50μm. GAPDH was served as internal control. N = 7 per experimental group. All data are expressed as mean ± SEM *P < .05, **P < .01 compared with WT sham, ^#^ *P* < .05, ^##^ *P* < .01 compared with WT MI

Further ex vivo study showed that HSF1 TG inhibited the increased HW/BW (Figure 5A), LVW/TL (Figure 5B), LW/TL (Figure 5C) and CSA of cardiomyocytes (Figure 5D, E) compared to WT mice 1 week after LAD ligation. Similarly, the abnormal reprogramming of *ANP* and *BNP* in WT MI mice were significantly rectified in HSF1 TG mice (Figure 5F). The infarct area of the mouse hearts in HSF1 KO mice was similar compared to WT mice 1 week after MI (Figure 5G). The myocardial apoptosis were both significantly and similarly increased in the MI mice in both Wt and HSF1 TG mice (Figure 5H, I). Together, these in vivo data from HSF1 TG mice further demonstrated that HSF1 is able to inhibit the pathological cardiac hypertrophy and promote the cardiac dysfunction induced by MI.

## 4 DISCUSSION

Although the long‐held view has been that cardiac hypertrophy in response to pathological stress including ischaemia is an adaptive response required to sustain cardiac function after loss of working cardiomyocytes, this conception has been challenged by evidence that MI‐induced LVH consistently damaged the compliance of the hearts and aggravated myocardial ischaemia.^15^ Accumulating evidence from studies in patients and animal models indicated that cardiac hypertrophy after MI is not a compensatory but rather a maladaptive process.^16^ Cardiac hypertrophy is not only a predictor but also a mediator of cardiovascular events predisposing patients to arrhythmias and heart failure.^17^ Therefore, prevention of cardiac hypertrophy is considered as a novel strategy in the treatment of MI.^18^ In particular, in the acute stage of the MI, the fatal arrhythmia ranks as one of the top cause of sudden death.^19^, ^20^ Therefore, it is very important to diminish the underlying pro‐arrhythmic hypertrophy after acute MI. Our group, together with the others, previously reported that HSF1 plays an important role in the myocardial protection against cardiac overload.^5^, ^21^ Although the beneficial effects of HSF1 were largely attributed to the decreases of myocardial fibrosis in long‐term of biochemical stress such as pressure overload and ischaemia/reperfusion injury,^6^ the cellular and molecular mechanism of the protective role of HSF1 in the pathological cardiac remodelling led by direct ischaemia is still undetermined.

To our knowledge, this is the first report that the endogenous cardioprotective factor, HSF1, plays an essential role in the development of cardiac hypertrophy occurred in the early stage of MI. In the present study, we have 3 important findings. First, we identified HSF1 as an essential mediator of cardiac hypertrophy induced by hypoxic stimuli. Second, we found that the phosphorylation of HSF1 is required for the preservation of cardiac morphology and function in the early stage of MI in vivo. Third, the underlying mechanism by which HSF1 attenuated ischaemia‐induced cardiac hypertrophy is associated with the blockage of the JAK2/STAT3 pathway. These data strongly suggested that maintaining of HSF1 at a higher level could prevent the occurrence of maladaptive cardiac hypertrophy and preserve the adaptation of the heart in response to ischaemia. Our findings indicated that HSF1 is critically involved in the myocardial hypertrophy evoked by MI, implying a promising therapeutic potential of HSF1 in MI patients.

HSF1 was demonstrated as a key factor involved in the adaptive mechanism of transition from adaptive cardiac hypertrophy to maladaptive heart failure,^6^ as well as an essential intrinsic factor protecting the cardiomyocytes against ischaemic injury via interacting with STAT3.^5^, ^7^ In the present study, our first finding is that HSF1 phosphorylation was inhibited by the ischaemic stimulation. We observed that the phosphorylation level of HSF1 was reduced in the MI‐induced cardiac hypertrophic infarct border zone area in vivo and hypoxia‐treated NRCMs in vitro. On the basis of these observations, we concluded that the phosphorylation of HSF1 is a potential mechanism of the cardiac hypertrophy occurred in the early stage of MI. We also postulated that ischaemia can restrain the activation of HSF1 activation.

Although it is an adaptive change to maintain cardiac contractile function after acute MI, the hypertrophic response to the ischaemia is a potential attributor of the fatal arrhythmia in MI patients.^22^ Here, we revealed that the deficiency of HSF1 exaggerated ischaemia‐induced cardiac hypertrophy and dysfunction. Loss of HSF1 augmented hypertrophic response of CMs to ischaemia, indicating that the reduction of HSF1 activity may promote the progression of cardiac hypertrophy and the transition to heart failure in vivo. The preservation of cardiac morphology and function was not attributed to the decrease in the myocardial death evidenced by a similar myocardial apoptosis between the WT mice and HSF1 KO mice 1 week after the LAD ligation. We also observed a deteriorated myocardial hypertrophic response to ischaemia in the HSF1‐deficient NRCMs in vitro. Finally, we revealed that enhanced HSF1 phosphorylation notably mitigated the pathological cardiac hypertrophy and inhibited the heart failure in the early stage of MI. These findings together indicated that HSF1 activation is critically involved in the cardiac hypertrophic response to ischaemia.

Abundant studies have implicated multiple signalling pathways in the progression of pathological cardiac hypertrophy after MI. However, effective therapies for antagonizing this process have not yet been developed. Accumulating evidence has indicated that JAK2/STAT3 pathway is a key mediator of cardiac hypertrophy.^9^ Deactivating JAK2 attenuates the cardiac dysfunction in mice after MI or ischaemia/reperfusion injury.

STAT3 is the most important effector of the JAK2 in the initiation and development of cardiac hypertrophy.^9^ After phosphorylation, STAT3 translocates from the cytoplasm to the nucleus and promotes the transcription of multiple hypertrophic factors including ANP and BNP.^23^Therefore, it is worthy to examine whether the expressions/activities of STAT3 are interfered by HSF1 in hearts upon acute ischemia. Our study showed that as an essential downstream transcription factor of JAK2, STAT3 mediates JAK2 signalling to regulate cardiac hypertrophic response to ischaemic stimulations. Upon MI, phosphorylated STAT3 was increased in the HSF1 KO mice, whereas inhibition of STAT3 phosphorylation ameliorated the ischaemia‐induced cardiac hypertrophy and cardiac dysfunction in mouse. These data suggested that HSF1 regulated the phosphorylation of JAK2 and consequently modulated the activation of STAT3. Although our results might not exclude other mechanisms by which HSF1 suppresses cardiac hypertrophy induced by ischaemia, the inhibitory effect of HSF1 on the development of cardiac hypertrophy seems to be largely dependent on the repression of JAK2/STAT3 by HSF1.

It is well known that STAT3 participates in a wide variety of physiological processes and directs seemingly contradictory responses such as proliferation and apoptosis. It was reported that cardiotrophin‐1 induces cardiac myocyte hypertrophy in part by up‐regulation of a local renin‐angiotensin system through STAT3 activation.^24^ Mice subjected to TAC alone developed concentric hypertrophy that accompanied activation of the components of the JAK/STAT signalling pathway manifested by an increase in phosphorylation of Jak2 and STAT3.^9^ Chronic activation of angiotensin II receptor 1 (AT1R) induced unregulated expression of the STAT3 gene, leading to nuclear accumulation of unphosphorylated form of STAT3, which significantly correlated with progression of cardiac hypertrophy.^10^ Mechanical stretch activates the JAK/STAT pathway in rat cardiomyocytes in vitro.^25^ Therefore, various researches indicated that the activation of STAT3 was able to provoke cardiac hypertrophy both in vivo and in vitro. While some other data showed that STAT3 is a protective factor against ischaemia or I/R injury, STAT3 was fund to be crucial in cardiomyocyte resistance to inflammation and other acute injury and in pathogenesis of age‐related heart failure.^26^ STAT3 was also essential for postnatal capillary vasculature maintenance in the heart which protected heart from ischaemic injury and heart failure.^27^ We ever reported a that granulocyte colony‐stimulating factor stimulates phosphorylation at Y705 and association of Stat3 with HSF1 and therefore enhances transcriptional activity of HSF1, leading to the cardio‐protection against I/R injury.^7^ Based on these finding, it can be postulated that STAT3 seemingly played a protective role in the cardiomyocytes against ischaemia or I/R injury; however, its role in the regulation of cardiac hypertrophy induced by ischaemia or MI remained unclear, although most of the evidence supported that the activation of STAT3 promoted cardiac hypertrophy under pressure overload. The confusing role of STAT3 in the regulation of cardiac hypertrophy might be blamed to the different types of animal model or in vitro cells. The duration of the treatment also changes the STAT3 phosphorylation level and sites. Moreover, the activation of different phosphorylated site of STAT3, such as S727 and Y705, may lead to different downstream effect.^28^ The JAK/STAT activation induced by angiotensin II is biphasic and time dependent in rat cardiomyocytes.^29^ Our findings revealed a detrimental role of STAT3 in the ischaemia‐related cardiac hypertrophy, which is not consistent with our previous report. The following reasons may contribute to the divergences. First, we used different animal model. The present study discussed the regulation of HSF1 by STAT3 in an early stage of MI but not I/R injury in mice. Second, the present study detected the phosphorylation of STAT3 at S727 but not the Y705. The phosphorylation of these 2 different sites leads to different results.

In summary, our study provides insights into the mechanisms of ischaemia‐induced cardiac hypertrophy and may have significant implications for the development of strategies for the treatment of MI through targeting of the HSF1. The present study brought a novel tragedy that controlling cardiac hypertrophy induced by cardiac ischaemia through regulation of HSF1. We provided evidence illustrating that the underlying cardioprotective mechanism of HSF1 is dependent of JAK2/STAT3 pathway. The present study indicated a potential target for pharmacological treatment of cardiac hypertrophy after MI in patients.

## CONFLICT OF INTERESTS

The authors confirm that there is no conflict of interest.
