# Supplementary material for: Heat‐shock transcription factor 1 is critically involved in the ischaemia‐induced cardiac hypertrophy via JAK2/STAT3 pathway
Source: J Cell Mol Med. 2018 Jul 11;22(9):4292–303. doi: 10.1111/jcmm.13713 (PMC6111827; doi:10.1111/jcmm.13713)
Supplement: Supplementary file 1 [file JCMM-22-4292-s001.pdf]

## Supplementary Data

**Deficiency of heat shock transcription factor 1 deteriorated ischemia-induced cardiac hypertrophy via JAK2/STAT3 pathway**

Lingyan Yuan[1*], Lin Qiu[2*], Yong Ye[3], Jian Wu[3], Shuchun Wang[4], Xingxu Wang[3], Ning Zhou[5#], Yunzeng Zou[3#]

1. Department of kinesiology, Institute of physical education, Shanghai Normal University, Shanghai 200234, China.
2. Department of Pharmacy, Tongji Hospital, Tongji Medical College, Huazhong University of Science and Technology, Wuhan, 430030, China
3. Shanghai Institute of Cardiovascular Diseases, Zhongshan Hospital and Institutes of Biological Science, Fudan University, Shanghai, 200032, China
4. Department of Computer Tomography and Magnetic Imaging, Yidu Central Hospital, Weifang Medical College, Weifang, 430030, China
5. Division of Cardiology, Department of Internal Medicine, Tongji Hospital, Tongji Medical College, Huazhong University of Science and Technology, Wuhan, 430030, China

[*] These two authors contributed equally to this work

**Correspondence to:**

Ning Zhou, M.D., Ph.D.    Email: zhouning@tjh.tjmu.edu.cn

Yunzeng Zou, M.D., Ph.D.    E-mail: zou.yunzeng@zs-hospital.sh.cn

## Supplementary Tables

Supplementary Table 1: A list of primers used in this study.

| Genes | Forward primer (5′--3`) | Reverse primer (5′--3`) | GenBank Accession |
|---|---|---|---|
| Rat | | | |
| *ANP* | CCTGGACTGGGGAAGTCAAC | ATCTATCGGAGGGGTCCCAG | NM_012612.2 |
| *BNP* | TGACGGGCTGAGGTTGTTTT | ACACTGTGGCAAGTTTGTGC | NM_031545.1 |
| *GAPDH* | TCTCTGCTCCTCCCTGTTCT | ACCAGCTTCCCATTCTCAGC | NM_017008.4 |
| Mouse | | | |
| *ANP* | GCTTCCAGGCCATATTGGAG | GGGGGCATGACCTCATCTT | NM_008725 |
| *BNP* | GAGGTCACTCCTATCCTCTGG | GCCATTTCCTCCGACTTTTCTC | NM_008726 |
| *GAPDH* | AATGGATTTGGACGCATTGGT | TTTGCACTGGTACGTGTTGAT | NM_008085 |

## Supplementary Figure 1

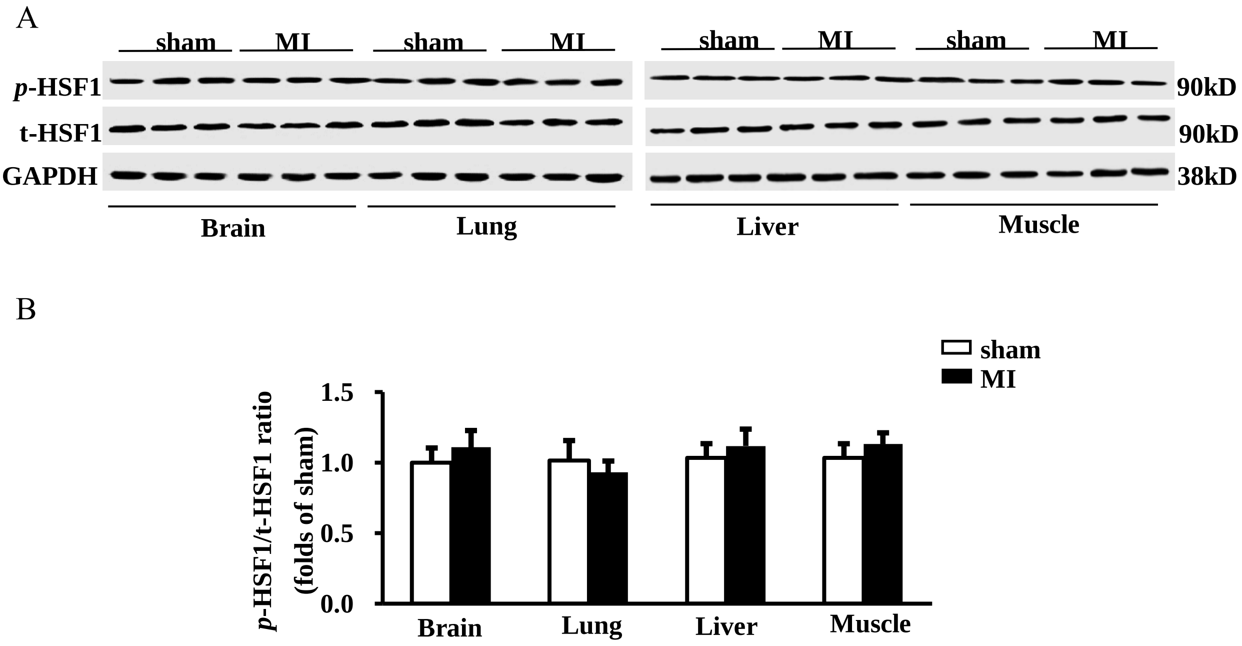


Figure legend

Figure 1. **The expression and phosphorylation of HSF1 in the mouse organs.** (A). Representative western blots of total and phosphorylation level of HSF1 in mouse organs. (B). Quantitative results of total and phosphorylation level of HSF1.
